# Differences in Body Mass Index Trajectories and Their Classification, Sociodemographic Characteristics, and Health Behaviors between People with and without Disabilities Using Korea Health Panel Survey Data

**DOI:** 10.3390/ijerph19052827

**Published:** 2022-02-28

**Authors:** Yea-Li-A Song, Jae-Hyun Park

**Affiliations:** 1 Medical Research Institute, Sungkyunkwan University School of Medicine, Suwon 16419, Korea; ylasong@naver.com; 2 Department of Social and Preventive Medicine, Sungkyunkwan University School of Medicine, Suwon 16419, Korea

**Keywords:** body mass index, trajectory, physical disability, disability, brain lesion disorder

## Abstract

A high body mass index (BMI) is an important factor that negatively affects the health of people with disabilities. In particular, since the high BMI has a cumulative effect on the occurrence of complications such as cardiovascular disease, it is required to investigate the data through longitudinal studies rather than cross-sectional studies. Therefore, we conducted a longitudinal follow-up study to examine the differences in the BMI trajectories of people in South Korea with disabilities, as well as the sociodemographic characteristics and health behaviors that classify individual trajectories into clusters. Participants aged 40 to 79 years who responded to the Korea Health Panel Survey (KHPS) from 2009 to 2018, 283 people with physical disabilities or brain lesion disorders, and 849 people without disabilities, were extracted. We found that the differences in the initial BMI between clusters were larger in 60–79-year-old people with disabilities (men 22.5 kg/m^2^, 26.9 kg/m^2^; women 23.8 kg/m^2^, 28.1 kg/m^2^) than in those without disabilities (men 22.1 kg/m^2^, 23.3 kg/m^2^; women 24.8 kg/m^2^, 25.6 kg/m^2^). Also, logistic regression analysis showed that, among the people with disabilities, women (OR = 1.94), those who lived alone (OR = 2.36), and those who were economically inactive (OR = 1.78) were more likely to be classified into the higher BMI category than those who were not. To effectively manage the BMI, it would be better to focus on women with disabilities, people with disabilities living alone, and people who are economically inactive.

## 1. Introduction

Anyone can experience a disability, but it is especially common among the elderly in South Korea [1]. The prevalence of people with disabilities at home increased with age, at 7.46% for those aged 55–59 and 13.68% for those aged 65–69, showing a sharp increase among elderly people aged 65 and over. Obesity and diseases such as diabetes, high cholesterol, and hypertension were reported to occur more often in people with disabilities than without [2,3,4]. Among these diseases, it is suggested that obesity has a large effect on health. One study [5] reported that people with physical disabilities had 1.96 times the healthcare costs of people without physical disabilities. It was also reported that obese people with physical disabilities had 1.13 times the healthcare costs of normal-weight people with physical disabilities, and 2.2 times that of normal-weight people without physical disabilities. Accordingly, obese people may use medical services and spend more on medical expenses than people of normal weight.

The body mass index (BMI), which is measured as weight relative to height squared, has been used in several studies as an indicator of obesity, but most studies analyzed BMI values at one point in time. However, more useful information can be obtained by analyzing repeated measurements over multiple time points. Analyzing the BMI trajectory is a way to examine the changes in values over time due to the accumulated effects of various factors. In this way, it is possible to assess the health status of the target population more accurately and provide more appropriate interventions for each type of change. Therefore, to explore the BMI, a longitudinal follow-up study that analyzes data from multiple time points rather than one time point seems more appropriate. However, previous studies rarely investigated the BMI trajectories of people with disabilities. In one study [6], the prevalence of BMI and its differences by year were analyzed, but it did not explore individuals’ BMI changes or differences between groups by similar patterns of change.

Although several studies have examined BMI changes in adults over time [7], no studies have investigated the difference in BMI trajectories between people with and without disabilities. In addition, studies on the BMI of people with and without disabilities were insufficient to analyze the changes in individual BMI trajectories or contributing factors that caused individual trajectories to be classified into risk clusters. Therefore, in this study, people with and without disabilities were extracted from representative cohort data of South Koreans and analyzed for comparison, and BMI trajectories were also analyzed for a longitudinal follow-up study. The objective of this study was to identify distinct BMI trajectories between people with and without disabilities. Furthermore, we aimed to examine the differences between the trajectories and elucidate the sociodemographic characteristics and health behaviors associated with those trajectories.

## 2. Materials and Methods

This study used the Korea Health Panel Survey (KHPS) data to analyze the BMI trajectories of people with and without disabilities for 10 years from 2009 to 2018.

### 2.1. Data

In this study, data (version 1.7) from the second through to the thirteenth waves (2009–2018) of the KHPS were used. The KHPS has been conducted annually since 2008 by the Korea Institute for Health and Social Affairs and the National Health Insurance Service for the same subjects [8]. The KHPS data are national-scale data on the use of healthcare services, healthcare expenditures, healthand-welfare-related indices, etc. By using 90% of the 2005 Population and Housing Census data as a sampling frame, national representation was maintained. The Population and Housing Census is a survey conducted in 20% of all households every five years in South Korea [9]. For the KHPS, sample households were extracted using a probability proportionate and stratified cluster sampling method. In the first survey in 2008, 7866 households and 24,616 respondents were surveyed. After that, until the 13th survey in 2018, the sample size was reduced to 4232 households due to death or refusal to survey, and the retention rate of the original sample was 53.8%.

### 2.2. Study Population

**Figure 1:**
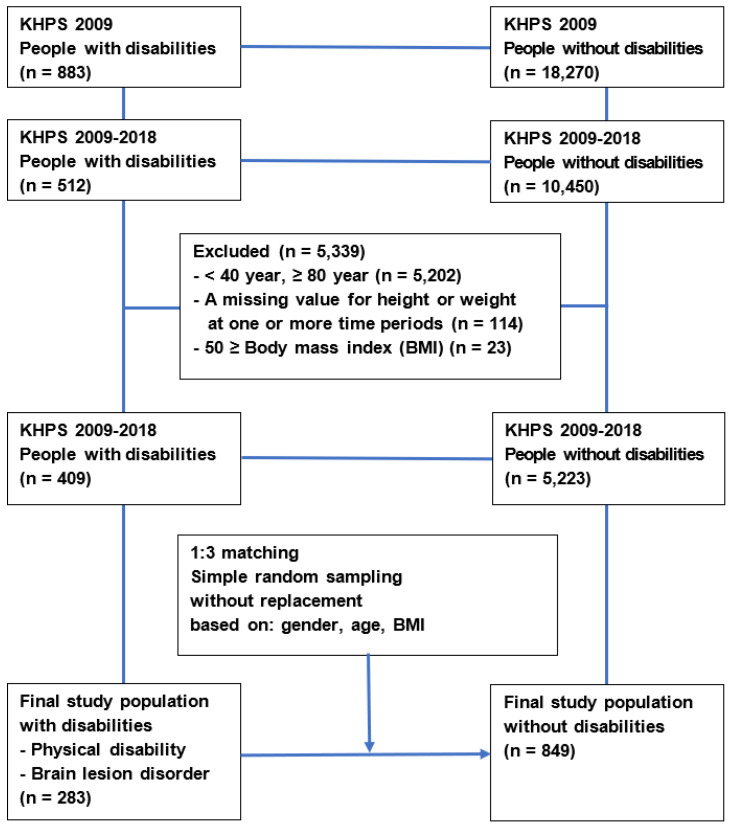
Flow diagram of subject selection from the Korea Health Panel Survey (KHPS) data.

The subjects who participated in the 2009 KHPS included 883 people with disabilities (4.6%) and 18,270 (95.4%) without disabilities (Figure 1). Among them, 512 people with disabilities and 10,450 without disabilities who participated in the KHPS from 2009 to 2018 were considered for this study. We excluded subjects who were under 40 years old or over 80 years old (*n* = 5202), had a missing value for weight or height at one or more time points (*n* = 114), and a BMI of over 50 (*n* = 23). For this study, first, all the people with a physical disability and brain lesion disorder in the data prepared to the previous stage (*n* = 409) were extracted (*n* = 283). A physical disability was defined as a disability related to amputation, motor disturbance, joint disability, deformity of the limbs, and spinal cord injury [10,11]. Brain lesion disorders were caused by stroke, brain damage, and brain palsy. We also randomly extracted three times as many people without disabilities as the number of people with disabilities by gender, age group (40–59 years old and 60–79 years old), and BMI group (~18.4, 18.5–22.9, 23.0–24.9, and 25.0 kg/m^2^). Among the selected women with and without disabilities, we intended to exclude pregnant women between 2009 and 2018, but no such subjects were found. Finally, the study population included 283 people with disabilities and 849 without disabilities.

### 2.3. Measurements

The dependent variable BMI was calculated as weight (kg)/height (m^2^) using self-reported weight and height. In addition, as the independent factors, we used sociodemographic characteristics (living alone, education, current economic activity, adjusted income, and type of health insurance) and health behaviors (heavy drinking and current smoking). The variable for living alone was grouped into two categories of yes and no. The respondents’ education, based on graduation, was divided into two categories (less than middle school and more than high school). The variable for current economic activity was divided into two categories (worked for the purpose of income and did not). For the variable for adjusted income, first, the total income of a household was divided by the square root of the number of household members, and then this value was divided into the lowest first and second to fifth quintiles. The type of health insurance was classified into national health insurance and medical aid.

If a man drank more than 7 glasses of alcohol and a woman drank more than 6 glasses of alcohol in one session for at least 8 days in the past 30 days, it was defined as heavy drinking. Current smoking was divided into two groups of yes and no. The study set gender, age, and residence area as confounding factors. Ages were classified into 40–59 and 60–79 year age groups [12,13]. The areas of residence were divided into two groups. Special cities and metropolitan cities were classified into the large city group, and other small-sized cities and farming and fishing villages were classified as small city and rural groups, referring to the administrative units of South Korea.

### 2.4. Analysis

In this study, the classification method suggested by Leffondre’ et al. [14] was used to describe and classify changes in BMI over 10 years. First, 24 measurements were calculated to describe the characteristics of the subjects’ changes in BMI. Next, factor analysis was conducted to extract the measurements that could best show the change patterns of the BMIs among the 24 measurements. Finally, we performed a cluster analysis to classify the individuals’ BMI change patterns into respective subgroups.

To determine the most appropriate number of clusters, analysis was performed by entering the number of clusters from 2 to 7. The final number of clusters was determined to be 2 by considering the research purpose and referring to the pseudo F statistic and the Cubic Clustering Criterion (CCC). The maximum pseudo-F statistic indicates the most suitable number of clusters, and a CCC of 2 or higher indicates that the identification is reliable. In addition, each cluster was set to include more than 5 subjects. Differences in the distribution of sociodemographic characteristics and health behaviors between the two BMI clusters (lower BMI and higher BMI clusters) were tested using the chi-squared test for categorical data. To determine whether sociodemographic characteristics and health behaviors had different influences on the BMI clusters of subjects with and without disabilities, we used the multiple logistic regression model. For this analysis, we adjusted for confounding factors such as gender, age, and residence area. Statistical significance was defined as a 2-tailed *p*-value of less than or equal to 0.05. R version 3.6.3 (R Foundation for Statistical Computing) was used to analyze the data (Appendix A).

## 3. Results

### 3.1. BMI Trajectories for All Study Subjects

**Figure 2:**
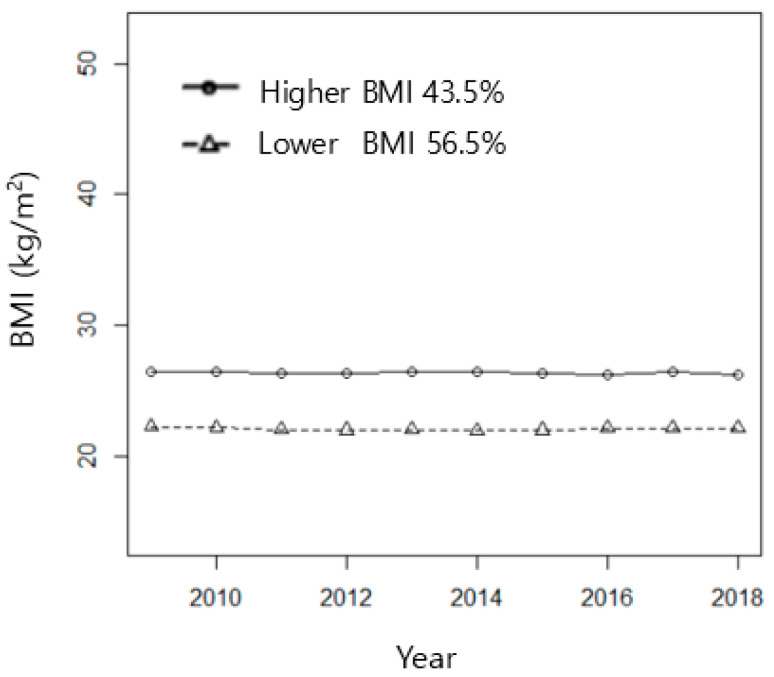
BMI clusters of all study subjects, including people with and without disabilities (cluster means).

All individual trajectories were classified into two BMI clusters (Table 1 and Figure 2), which were then used in chi-squared analysis and logistic regression analysis. The clusters were defined as higher BMI (baseline BMI = 26.5 kg/m^2^, *n* = 492, and 43.5%) and lower BMI (baseline BMI = 22.3 kg/m^2^, *n* = 640, and 56.5%), consistent over 10 years.

### 3.2. BMI Trajectories by Subgroup According to Disability, Gender, and Age

**Figure 3:**
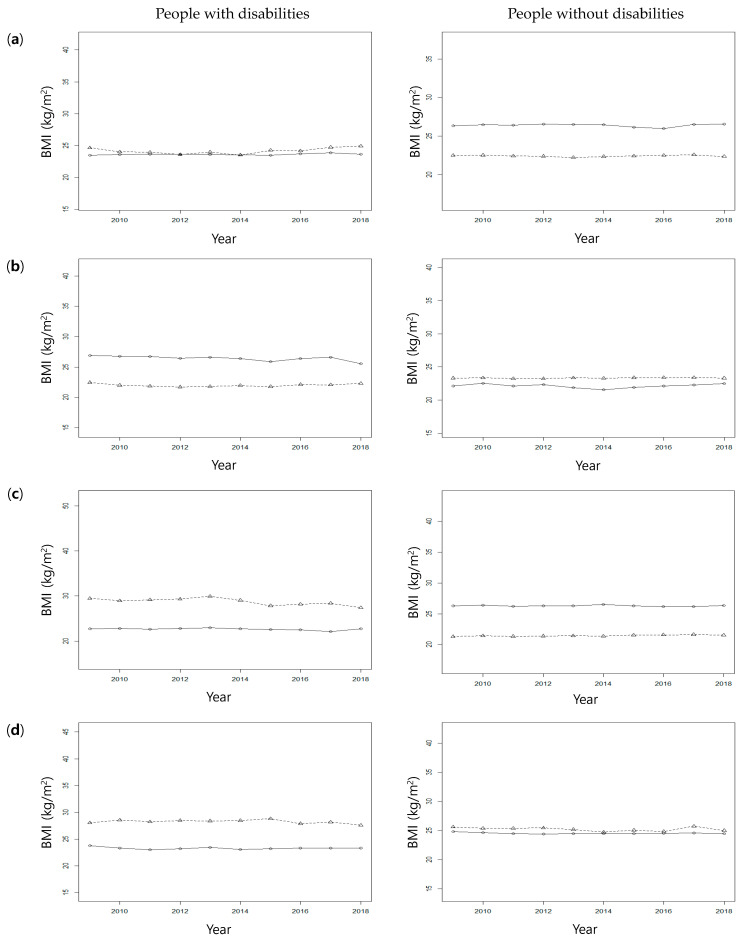
BMI clusters by disability, gender, and age (cluster means). (**a**) Men aged 40–59 years, (**b**) men aged 60–79 years, (**c**) women aged 40–59 years, and (**d**) women aged 60–79 years.

Next, the classification method was applied to each subgroup according to disability, gender, and age to determine the differences in BMI trajectories for each group. The analysis showed that the BMI clusters were generally stable without significant changes over time (Table 2 and Figure 3). Among the people without disabilities, the difference in the average BMI for each cluster was relatively large in people aged 40 to 59 (men, lower BMI 22.5 kg/m^2^, higher BMI 26.3 kg/m^2^; women, lower BMI 21.3 kg/m^2^, higher BMI 26.3 kg/m^2^), and it was relatively small in those aged 60–79 (men, lower BMI 22.1 kg/m^2^, higher BMI 23.3 kg/m^2^; women, lower BMI 24.8 kg/m^2^, higher BMI 25.6 kg/m^2^). In contrast, among the people with disabilities, the difference in the average BMI was relatively large in the 60–79-year-old groups (men, lower BMI 22.5 kg/m^2^, higher BMI 26.9 kg/m^2^; women, lower BMI 23.8 kg/m^2^, higher BMI 28.1 kg/m^2^).

### 3.3. BMI Clusters by Sociodemographic Characteristics and Health Behaviors

The number and percentage of characteristics in people with and without disabilities are listed in Table 3 by clusters. Among subjects with disabilities, people belonging to the lower BMI cluster (*n* = 161, 56.9%) were more numerous than those belonging to the higher BMI cluster (*n* = 122, 43.1%). Among them, those who were women (*n* = 69, 53.1%), lived alone (*n* = 21, 65.6%), and were not economically active (*n* = 78, 50.3%) were more likely to be included in the higher BMI cluster. The analysis results for subjects without disabilities were similar to those of the subjects with disabilities. The subjects without disabilities who were women (*n* = 206, 52.8%), lived alone (*n* = 40, 57.1%), had a lower level of education (*n* = 238, 48.9%), and were not economically active (*n* = 156, 51.0%) were more likely to be included in the higher BMI cluster, and those who were smoking (*n* = 58, 27.2%) were less likely to be classified into the higher BMI cluster than those who did not (*n* = 306, 49.0%).

### 3.4. According to Sociodemographic Characteristics and Health Behaviors, Trajectories Were Classified into BMI Clusters

Logistic analysis was performed to determine the differences in the influencing factors that classified each person with and without disabilities into each cluster (Table 4). Among the people with disabilities, gender, living alone, and current economic activity were significantly associated with the patterns of the BMI clusters. Logistic regression analysis revealed that, among the people with disabilities, those who were women (odds ratio (OR) = 1.94, 95% confidence interval (CI): 1.07–3.53), lived alone (OR = 2.36, 95% CI: 1.01–5.55), and were not economically active (OR = 1.78, 95% CI: 1.04–3.07) were more likely to be included in the higher BMI category than those who were not. Among the people without disabilities, those who had lower education (OR = 1.54, 95% CI: 1.11–2.15) and were not economically active (OR = 1.41, 95% CI: 1.02–1.95) were more likely to be included in the higher BMI category than those who were not. Those who were smokers were less likely to be included in the higher BMI category (OR = 0.46, 95% CI: 0.31–0.68) than those who were not. Associations between education or current smoking and BMI clusters were not seen among the people with disabilities.

## 4. Discussion

The results of this study showed that the differences in the average BMI for each cluster of subgroups according to disability, gender, and age were less in 60–79-year-old people than in 40–59-year-old people among those without disabilities. However, among people with disabilities, these differences were relatively large. Compared with people without disabilities, the BMI trajectory of people with disabilities with poorly managed weight may be more problematic. In other words, people who do not manage their weight well may develop more cardiovascular complications than those who do, and this may be a bigger problem in people with disabilities than without.

The people with disabilities analyzed in this study had physical disabilities or brain lesions. Previous studies [15] suggested that physiological changes, energy metabolism, and muscle atrophy were factors affecting their weight gain. In addition, basic care such as eating and physical activity is required to properly manage weight. The inability of a person to carry out his or her daily activities without the help of others can be detrimental to their health [16]. However, people with disabilities often have difficulties performing daily activities on their own. As such, they may also have significant restrictions on activities outside their living environment, such as bringing in groceries or preparing meals at home [17]. According to a survey [1] on the status of people with disabilities in South Korea, many people with physical disabilities (25.5%) and brain lesions (77.0%) responded that they needed support for preparing meals, and among people with disabilities, 87.6% answered that they needed rehabilitation exercises and physical education. Also, several studies [18,19] in other regions reported that these needs of people with disabilities were often not met.

People with disabilities were reported not to engage in the level of physical activity necessary for good health [15]. The reasons for this may include pain, limitations in physical functions required for activities [20], and difficulty in accessing an environment where exercise can be performed. Therefore, it is thought that these factors can lead to obesity due to an imbalance in which the energy intake is greater than the energy consumption. In addition, the low socioeconomic status of many people with disabilities may have influenced their BMI. Low income can make it difficult to access high-quality food [17]. Thus, there may be restrictions on the food choices that people with disabilities want or need. Previous studies have shown a relationship between low socioeconomic levels and obesity, and the BMI gap between groups may have grown over time [13,21].

As a result of the logistic regression analysis of people with disabilities, women were more likely to be included in the higher BMI cluster than men. No studies were found on gender differences in obesity in people with disabilities. Instead, in studies on the whole population, adult women were more likely to be obese than adult men. Health behaviors, that is, eating habits and physical activity, and social factors were mainly suggested as the causes. Eating habits are behaviors that show distinct gender differences, and it has been reported that women tended to eat more healthy foods and consume more sugary foods than men [22]. In addition, several previous studies suggested that women were less physically active than men [23,24], and that there may be restrictions on physical activity depending upon gender due to social norms or expectations [25]. In addition, it is necessary to consider the reproductive role of women in relation to BMI. Insulin resistance may increase during pregnancy, and thus, pregnancy may be a risk factor for obesity [26].

Unlike women without disabilities, there were significantly more women with disabilities classified into the higher BMI cluster than men with disabilities. This finding may have been influenced by the low employment rate of women with disabilities. The employment rate of people with disabilities is 36.9%, which is lower than the 61.3% employment rate of the total population, and even among people with disabilities, the employment rate of women with disabilities (23.4%) is only about half that (47.0%) of men with disabilities [1]. It is thought that the poor economic status due to the low employment rate may have had an effect on the increase in BMI. This is consistent with the results of previous studies that women who had been unemployed for a long time, unlike men, had an increased risk of becoming obese [27,28], and that economic difficulties were associated with weight gain [13,29].

Also, people with disabilities living alone were more likely to be classified into the higher BMI cluster than those who did not live alone. This result suggests that living alone has a greater effect on weight management in people [30] with disabilities than without. Some people with disabilities have difficulties performing daily activities such as eating and physical activity on their own, which may have contributed to the increase in BMI. These problems can be solved with the help of others, but many people with physical disabilities (25.2%) and brain lesions (8.6%) have no one to help them in their daily lives [1]. Even for people with disabilities, the range of normal activities varies depending upon the type and degree of each person’s disability. However, even people with disabilities who are able to do activities on their own to some extent cannot receive the positive effects of living together if they live alone. Living with family or other people can provide several health benefits, including controlling health behaviors and providing mutual social support [31]. Living with other people also increases the household income and allows them to benefit from economies of scale, which, in turn, provides more sufficient access to resources conducive to health [32].

In contrast, it may be difficult for people at lower socioeconomic levels for education and occupation to marry or live together. A survey [1] of people with disabilities in South Korea found that health and disability problems accounted for a high percentage of the reasons for not marrying people with physical disabilities (27.5%) or brain lesions (51.9%). Therefore, it seems that people with disabilities may have more difficulties getting married or living together by forming relationships with other people than those without disabilities.

The results of this study showed that both people with and without disabilities were often included in the higher BMI cluster if they were not currently economically active, which is consistent with the results of previous studies [28], suggesting a relationship between unemployment status and high BMI. However, it is necessary to examine the relationship between economic activity and health behaviors that can affect weight, such as eating, physical activity, and sleep. In economically difficult situations, people seem to be more likely to choose cheap foods that are high in calories and low in quality [33,34]. It was also suggested that unemployment may decrease physical activity [35] and sleep quality [36,37], and sleep deprivation affects weight gain through metabolic and endocrine changes [38,39].

According to the results of this study, the relationship between education and the BMI of people with disabilities was not significant. Previous studies [12,40,41] reported that the BMI values of people with lower education fell within the higher range compared to people with higher education. However, studies on the mechanism of the effect of education level and the BMI of people with disabilities could not be confirmed. The study results showed that the effect of the education was not significant for people with disabilities compared to people without disabilities. This means that the effect of education level on health management is not significant for people with disabilities compared to those without disabilities. People without disabilities are more likely to be in good health if they have a high level of education. A higher level of education can enable people to have jobs with good conditions, such as a higher social position, and thereby increase their income and secure better resources conducive to health [42,43]. Education can also contribute to improving the knowledge, attitudes, and behaviors necessary for each person to manage their own health [32]. People with disabilities, even with a high level of education, are less likely to have good health than people without disabilities. Rather, it can be inferred that the type and severity of the disability, economic level, and living alone have a relatively greater influence on people with disabilities.

This study had several limitations. First, due to data limitation, we could not analyze people with other types of disabilities such as communication or intellectual disabilities. In addition, this study was conducted on people who had already developed a disability, and it was not possible to study recently acquired disabilities. In other words, it was not possible to compare before and after the onset of disability. Also, the cause and site of the disability could not be considered, so the difference in BMI according to these factors could not be examined. Nevertheless, in this study, people with and without disabilities were matched and analyzed from representative KHPS (2009–2018) data. In addition, we conducted a longitudinal follow-up study that analyzed the trajectory of the cohort data for 10 years, not a cross-sectional study at one time point. In addition, the BMI trajectories of people with and without disabilities and the contributing factors that made them belong to a risk cluster were compared and examined.

## 5. Conclusions

In conclusion, we found that the initial BMI differences between the two clusters (lower BMI, higher BMI) were larger in the 60–79-year-old group of people with disabilities compared with the same age group without disabilities. In particular, it was found that people with disabilities were less able to manage their weight if they were women, lived alone, and were not currently economically active. When considering the results of this study, it may be necessary to keep in mind that people with physical disabilities or brain lesions need some help with their daily lives. To solve this problem, it is necessary to provide customized healthcare services for people with disabilities who have more difficulty with weight management. In addition, future studies should include analysis of fat-free mass index and waist circumference to provide a better understanding of obesity.

## Figures and Tables

**Table 1:** BMI clusters of all study subjects, including people with and without disabilities.

			Intercept (BMI at Baseline)		Linear Slope	
	*n*	%	Mean	SD ^1^	Mean	SD
Lower BMI	640	56.5	22.3	1.91	−0.00	0.21
Higher BMI	492	43.5	26.5	2.16	−0.01	0.26

^1^ SD = standard deviation.

**Table 2:** BMI clusters by gender and age and differences between people with and without disabilities.

			People with Disabilities (*n* = 283)						People without Disabilities (*n* = 849)					
					Intercept (BMI at Baseline)		Linear Slope				Intercept (BMI at Baseline)		Linear Slope	
	Age	Cluster	*n*	%	Mean	SD	Mean	SD	*n*	%	Mean	SD	Mean	SD
Men	40–59	Lower BMI	49	69.0	23.5	2.58	0.02	0.13	130	61.0	22.5	1.88	0.00	0.18
		Higher BMI	22	31.0	24.7	3.94	0.06	0.37	83	39.0	26.3	1.96	0.00	0.21
	60–79	Lower BMI	63	76.8	22.5	1.98	0.01	0.19	5	2.0	22.1	1.58	0.00	0.00
		Higher BMI	19	23.2	26.9	2.87	−0.10	0.26	241	98.0	23.3	2.36	0.01	0.19
Women	40–59	Lower BMI	27	77.1	22.8	2.84	−0.04	0.26	54	51.4	21.3	2.09	0.03	0.20
		Higher BMI	8	22.9	29.5	6.54	−0.20	0.45	51	48.6	26.3	2.40	0.00	0.22
	60–79	Lower BMI	62	65.3	23.8	2.08	−0.02	0.22	232	81.4	24.8	2.61	−0.02	0.22
		Higher BMI	33	34.7	28.1	2.17	−0.05	0.34	53	18.6	25.6	2.65	−0.04	0.46

**Table 3:** BMI clusters by sociodemographic characteristics and health behaviors and differences between people with and without disabilities.

Variables			People with Disabilities							People without Disabilities					
		Total	Lower BMI		Higher BMI				Total	Lower BMI		Higher BMI			
		*n*	*n*	%	*n*	%	*X* ^2^	*p*-Value	*n*	*n*	%	*n*	%	*X* ^2^	*p*-Value
Total		283	161	56.9	122	43.1			849	479	56.4	370	43.6		
Gender	Men	153	100	65.4	53	34.6	9.003	0.003	459	295	64.3	164	35.7	24.357	0.000
	Women	130	61	46.9	69	53.1			390	184	47.2	206	52.8		
Age	40–59	106	57	53.8	49	46.2	0.484	0.487	318	190	59.7	128	40.3	2.080	0.149
	60–79	177	104	58.8	73	41.2			531	289	54.4	242	45.6		
Residence area	Large city	104	52	50.0	52	50.0	2.754	0.097	311	165	53.1	146	46.9	2.049	0.152
	Small city and rural	179	109	60.9	70	39.1			538	314	58.4	224	41.6		
Living alone	Yes	32	11	34.4	21	65.6	6.459	0.011	70	30	42.9	40	57.1	5.122	0.024
	No	251	150	59.8	101	40.2			779	449	57.6	330	42.4		
Education	Less than middle school	189	109	57.7	80	42.3	0.062	0.803	487	249	51.1	238	48.9	12.499	0.000
	More than high school	94	52	55.3	42	44.7			362	230	63.5	132	36.5		
Current economic	Yes	128	84	65.6	44	34.4	6.634	0.010	543	329	60.6	214	39.4	10.189	0.001
activity	No	155	77	49.7	78	50.3			306	150	49.0	156	51.0		
Adjusted income	1st quintile (lowest)	105	55	52.4	50	47.6	1.057	0.304	188	99	52.7	89	47.3	0.964	0.326
	2nd–5th quintiles	175	104	59.4	71	40.6			654	373	57.0	281	43.0		
Type of health	National health insurance	233	134	57.5	99	42.5	0.088	0.766	815	462	56.7	353	43.3	0.353	0.553
insurance	Medical aid	50	27	54.0	23	46.0			34	17	50.0	17	50.0		
Heavy drinking	Yes	25	14	56.0	11	44.0	0.000	1.000	111	68	61.3	43	38.7	1.002	0.317
	No	258	147	57.0	111	43.0			738	411	55.7	327	44.3		
Current smoking	Yes	61	40	65.6	21	34.4	1.845	0.174	213	155	72.8	58	27.2	29.653	0.000
	No	219	120	54.8	99	45.2			625	319	51.0	306	49.0		

**Table 4:** Effect of sociodemographic characteristics and health behaviors according to BMI cluster and differences between people with and without disabilities (Reference: Lower BMI).

Variables		People with Disabilities				People without Disabilities			
		OR	95% CI		*p*-Value	OR	95% CI		*p*-Value
Gender	Men	Ref				Ref			
	Women	1.94	1.07	3.53	0.029	1.20	0.84	1.72	0.324
Age	40–59	Ref				Ref			
	60–79	0.62	0.35	1.11	0.108	0.78	0.55	1.10	0.156
Residence area	Large city	1.32	0.78	2.24	0.298	1.20	0.89	1.62	0.228
	Small city and rural	Ref				Ref			
Living alone	Yes	2.36	1.01	5.55	0.049	1.47	0.85	2.55	0.166
	No	Ref				Ref			
Education	Less than middle school	0.81	0.44	1.50	0.504	1.54	1.11	2.15	0.010
	More than high school	Ref				Ref			
Current economic	Yes	Ref				Ref			
activity	No	1.78	1.04	3.07	0.037	1.41	1.02	1.95	0.039
Adjusted income	1st quintile (lowest)	1.25	0.71	2.22	0.440	0.98	0.68	1.41	0.897
	2nd–5th quintiles	Ref				Ref			
Type of health	National health insurance	Ref				Ref			
insurance	Medical aid	0.72	0.34	1.51	0.383	0.84	0.39	1.80	0.648
Heavy drinking	Yes	1.68	0.66	4.29	0.279	1.28	0.80	2.03	0.302
	No	Ref				Ref			
Current smoking	Yes	0.80	0.39	1.61	0.525	0.46	0.31	0.68	0.000
	No	Ref				Ref			

## Data Availability

Data were obtained from the Korean Institute for Health and Social Affairs (KIHSA) and are available with the permission of the KIHSA. The contact information is as follows: Korean Institute for Health and Social Affairs; https://www.khp.re.kr:444/eng/main.do (accessed on 1 August 2021); email: khp@kihasa.re.kr.

## Abbreviations

BMI	Body Mass Index
KHPS	Korea Health Panel Survey
SD	Standard Deviation

## Appendix A. Measures of Change

### Appendix A.1. Elementary Measures of Change (1–10)

1. Range; 2. mean-over-time, 3. standard deviation (SD), 4. coefficient of variation (CV), 5. change, 6. mean change per unit time, 7. change relative to the first score, 8. change relative to the mean over time, 9. slope of the linear model, and 10. proportion of variance explained by the linear model [14]

### Appendix A.2. Measures of the Nonlinearity and Inconsistency of Change (11–18)

11. Maximum of the first differences, 12. SD of the first differences, 13. SD of the first differences per time unit, 14. mean of the absolute first differences, 15. maximum of the absolute first differences, 16. ratio of the maximum absolute difference to the mean-over-time, 17. ratio of the maximum absolute first difference to the slope, and 18. ratio of the SD of the first differences to the slope.

### Appendix A.3. Measures Sensitive to Nonmonotonicity and Abrupt Short-Term Fluctuations (19–24)

19. Mean of the second differences, 20. mean of the absolute second differences, 21. maximum of the absolute second differences, 22. ratio of the maximum absolute second difference to the mean-over-time, 23. ratio of the maximum absolute second difference to the mean absolute first difference, and 24. ratio of the mean absolute second difference to the mean absolute first difference.
